# Adaptive rewiring of purine metabolism promotes treatment resistance in H3K27M-mutant diffuse midline glioma

**DOI:** 10.21203/rs.3.rs-3317816/v1

**Published:** 2023-09-11

**Authors:** Erik R. Peterson, Peter Sajjakulnukit, Andrew J. Scott, Caleb Heaslip, Anthony Andren, Kari Wilder-Romans, Weihua Zhou, Sravya Palavalasa, Navyateja Korimerla, Angelica Lin, Alexandra Obrien, Ayesha Kothari, Zitong Zhao, Li Zhang, Meredith A. Morgan, Sriram Venneti, Carl Koschmann, Nada Jabado, Costas A. Lyssiotis, Maria G. Castro, Daniel R. Wahl

**Affiliations:** University of Michigan; University of Michigan; University of Michigan; Massachusetts College of Pharmacy and Health Sciences; University of Michigan; University of Michigan; University of Michigan; University of Michigan; University of Michigan; University of Michigan; University of Michigan; University of Michigan; University of Michigan; University of Michigan; University of Michigan; University of Michigan; University of Michigan; McGill University; University of Michigan; University of Michigan; University of Michigan

**Keywords:** Diffuse midline glioma, H3K27M, Radiation therapy resistance, purine metabolism

## Abstract

**Background:**

Diffuse midline gliomas (DMG), including diffuse intrinsic pontine gliomas (DIPGs), are a fatal form of brain cancer. These tumors often carry a driver mutation on histone H3 converting lysine 27 to methionine (H3K27M). DMG-H3K27M are characterized by altered metabolism and resistance to standard of care radiation (RT), but how the H3K27M mediates the metabolic response to radiation and consequent treatment resistance is uncertain.

**Methods:**

We performed metabolomics on irradiated and untreated H3K27M isogenic DMG cell lines and observed an H3K27M-specific enrichment for purine synthesis pathways. We profiled the expression of purine synthesis enzymes in publicly available patient data and in our models, quantified purine synthetic flux using stable isotope tracing, and characterized the *in vitro* and *in vivo* response to *de novo* and salvage purine synthesis inhibition in combination with RT.

**Results:**

DMG-H3K27M cells activate purine metabolism in an H3K27M-specific fashion. In the absence of genotoxic treatment, H3K27M-expressing cells have higher relative activity of *de novo*synthesis and lower activity of purine salvage due to decreased expression of the purine salvage enzymes. Inhibition of *de novo* synthesis radiosensitized DMG-H3K27M cells *in vitro* and *in vivo*. Irradiated H3K27M cells adaptively upregulate purine salvage enzyme expression and pathway activity. Silencing the rate limiting enzyme in purine salvage, hypoxanthine guanine phosphoribosyl transferase (HGPRT) when combined with radiation markedly suppressed DMG-H3K27M tumor growth *in vivo*.

**Conclusions:**

H3K27M expressing cells rely on *de novo* purine synthesis but adaptively upregulate purine salvage in response to RT. Inhibiting purine salvage may help overcome treatment resistance in DMG-H3K27M tumors.

## Background

Diffuse midline gliomas (DMG) are pediatric high-grade gliomas that arise in midline structures of the brain including the thalamus, cerebellum, and pons(1–3). In 2016, the World Health Organization described a subtype of DMG that carries a driver missense mutation in the tail domain of Histone H3 that converts the 27th residue from lysine (K) to methionine (M), termed H3K27M(1, 4). Patients with DMG-H3K27M carry a dire prognosis with over 90% dying within 2 years of diagnosis(2). Treatment options are limited for patients with DMG-H3K27M tumors. Surgical resection is often unfeasible due to the eloquent function of the midline tissues in which they arise(2, 3, 5). DMG-H3K27M tumors also derive minimal benefit from systemic therapies(3, 5–7). Radiation therapy (RT) is currently the only treatment modality that provides meaningful benefit to patients and is the current standard of care(8–12). However, RT typically only extends patient survival by months, and tumors regrow within the high-dose radiation field(8, 12, 13). This suggests that DMG-H3K27M tumor cells can effectively adapt and resist RT, leading to regrowth. Simply increasing the RT dosage given is not feasible owing to limiting toxicity in normal tissue. Therefore, there is a critical need to selectively increase the sensitivity of DMG-H3K27Ms to RT. Cellular metabolism controls RT efficacy in various tumor types, including adult brain malignancies(14–17). The H3K27M mutation causes a global shift in the epigenome, leading to large-scale gene expression changes. This is characterized by global hypomethylation of the H3K27 residue in promoter regions of H3K27M cells which is accompanied by increased activating H3K27 acetylation marks(4, 18–23). These alterations cause metabolic shifts specific for H3K27M-expressing tumors(24). Such shifts included increased dependence on methionine and pyrimidine metabolism, along with classically cancer-co-opted pathways such as glycolysis and the TCA cycle(25–27). How and whether these H3K27M-driven metabolic changes confer radiation resistance to DMGs is unknown.

The importance of the H3K27M mutation and its profound effect on metabolism suggest that this mutation may specifically confer RT resistance in DMG-H3K27M tumors. Here, we identify purine metabolism as an H3K27M-specific metabolic vulnerability that governs the RT response. Cells can synthesize the two main classes of purines (adenylates and guanylates) *de novo* from metabolic building blocks or salvage them from pre-formed nucleobases. We find that H3K27M-expressing cells preferentially utilize *de novo* guanylate synthesis during unperturbed growth, likely due to increased expression of the enzymes utilized in *de novo* synthesis and decreased expression of those used in salvage. Inhibitors of *de novo* guanylate synthesis sensitize DMG-H3K27M models to RT *in vitro* and *in vivo* but do not cure tumors. Unexpectedly, we find that H3K27M cells upregulate guanylate salvage in response to RT, which likely mediates resistance to inhibition of *de novo* purine synthesis. Knockdown of the rate limiting enzyme in guanylate salvage greatly increases RT efficacy in orthotopic H3K27M xenografts. These findings indicate that guanylate salvage inhibition may be a promising strategy to overcome RT resistance in DMG-H3K27M.

## Methods

### Cell Lines and Tissue Culture

Patient-derived H3K27M-isogenic (H3K27M and H3K27M-KO) cell line pairs (DIPGXIII and BT245) were generous gifts from Dr. Nada Jabado (McGill University)(20) and were cultured in Tumor Stem Media (TSM) as previously described(19).

### DIPGXIII-GFP/LUC and DIPGXIII-GPF/LUC-shHPRT1 cell lines

DIPGXIII H3K27M-expressing cells were plated in TSM media containing polybrene transfection reagent and 10X ready-made lentiviral stock containing expression constructs for GFP and firefly luciferase (GFP/LUC) (Lenti-GF1-CMV-VSVG) or ready-made shHPRT1 viral vector stock (Santa Cruz Biotechnology Cat #: sc-40679-V). Cells were centrifuged and washed and expanded in 15cm plates 24hrs later. Successful transduction was confirmed by GFP expression or puromycin selection and loss of HGPRT protein.

### Western Blotting

H3K27M-isogenic cells were lysed in RIPA buffer containing protease (cOmplete, Roche) and phosphatase inhibitors (phoSTOP, Roche) on ice with mechanical disruption at the start of incubation. Lysates were clarified and protein concentration was determined using BCA assay (ThermoFisher). SDS-PAGE was performed using at least 12.5ug protein on 4–20% Tris-Glycine gels. Proteins were semi-dry transferred onto nitrocellulose for 1.5hrs at 24V. Membranes were blocked in non-fat dry milk in TBST before incubation with primary antibody overnight at 4C. Membranes were washed with TBST before incubation with secondary antibody. West Pico Plus (ThermoFisher) ECL reagent and X-Ray film were used to measure expression as previously described(16). Antibodies used: H3K27M (Cell Signaling, Cat#: 74829), H3K27me3 (EMD-Millipore, Cat#: 07-449), inosine monophosphate dehydrogenase 1 (IMPDH1) (Cell Signaling, Cat#: 35914), HPRT1 (Invitrogen, Cat#: PA5-22281), beta-Actin (Santa Cruz, Cat#: sc-47778).

### Histone Purification

Histone purification was performed as described previously(24). H3K27M isogenic cells (> 1.0×10^6) were suspended in 1mL hypotonic lysis buffer and lysed by mechanical shearing on a rotator at 4C. Nuclei were isolated and suspended in 0.4N H_2_SO_4_ overnight to precipitate histones. Samples were clarified and trichloroacetic acid was added dropwise to the histone-containing supernatant, which were mixed and incubated on ice. Histones were pelleted, washed with ice-cold acetone, and air-dried. Histones were resuspended in ddH2O. Protein content was quantified using a BCA assay.

### Steady-State Metabolomics

H3K27M-isogenic cells (2–3×10^6 cells/biological replicate) were plated in 10cm dishes in TSM media and were grown to be small neurospheres (approximately 2 days in culture). Media was changed 2hrs before treatment. Cells were irradiated with 4Gy RT using a Philips RT250 (Kimtron Medical) at a dose rate of approximately 2 Gy/minute at the University of Michigan Rogel Comprehensive Cancer Center Experimental Irradiation Shared Resource (Ann Arbor, MI). Cells were incubated for 2hrs at 37°C. One plate from each group was analyzed by BCA assay quantify protein for standardization. After incubation, all cells were collected in individual 15mL conical tubes and pelleted. Supernatants were removed and metabolites were extracted with ice-cold 80% methanol on dry ice. Pellets were resuspended and incubated for on dry ice. Samples were centrifuged to clarify the metabolite extract and supernatants were transferred to new 15mL tubes. This step was repeated twice to ensure clarity. Metabolite extracts equating to roughly 1000μg of protein were transferred to 1.5mL microcentrifuge tubes before drying in a speed vacuum centrifuge or under a nitrogen blower. Liquid chromatography/mass spectrometery (LC/MS) analyses were performed as previously described(28). Agilent MassHunter Quantitative Analysis B.09.00 QqQ software was used to integrate and quantitate areas (Agilent Technologies).

### Steady-State Metabolomics Data Analysis

Metabolite lists were trimmed to exclude metabolites that fell below the noise threshold. Remaining metabolite abundances were median centered. H3K27M isogenic cells were analyzed in pairs. All metabolites whose abundance changed by a Log2 FC of |0.15| following RT in either H3K27M or H3K27M-KO cells were selected and combined into a single list. These lists were ordered by the absolute value of difference in post-RT Log2FC between K27M and KO cells. The top 25 metabolites were selected for pathway enrichment analysis using MetaboAnalyst 5.0 where the top 10 most significantly represented pathways were selected(29). Statistical analyses and heatmap construction were performed using GraphPad Prism 9.0.

### Stable Isotope Tracing

H3K27M isogenic cells (> 4.0×10^6 cells/replicate) were plated in 10cm dishes in TSM and grown into small neurospheres. Approximately 30min before RT, cells were pelleted and washed and replated with TSM media with stable isotope tracer molecules replacing the naturally occurring metabolites (2mM 2,8-deuterium hypoxanthine (2D-Hpx) or 4mM ^15^N-glutamine [Cambridge Laboratories Inc, Cat#: DLM-8658 and NLM-557, respectively]). One replicate plate from each group was set aside and given unlabeled TSM media to act as unlabeled control samples. Untreated and irradiated cells (4Gy) were incubated at 37°C for 3hrs after RT at which point metabolites were extracted.

LC/MS Analysis was performed on an Agilent system consisting of an Infinity Lab II UPLC with a 10-port valve coupled with a 6545 QToF mass spectrometer (Agilent Technologies) using a JetStream ESI source in negative mode. Source parameters: Gas Temp: 250 °C, Gas Flow: 13 L/min, Nebulizer: 35 psi, Sheath Gas Temp: 325 °C, Sheath Gas Flow: 12 L/min, Capillary: 3500 V, Nozzle Voltage: 1500 V.

Chromatographic separation was performed on an Agilent ZORBAX RRHD Extend 80Å C18, 2.1 × 150 mm, 1.8 μm column with an Agilent ZORBAX SB-C8, 2.1 mm × 30 mm, 3.5 μm guard column. The column temperature was 35 °C. Mobile phase A consisted of 97:3 water/methanol and mobile phase B was 100% methanol; both A and B contained tributylamine and glacial acetic acid at concentrations of 10mM and 15mM, respectively. The column was backflushed with mobile phase C (100% acetonitrile, no additives) between injections for column cleaning.

The LC gradient was as follows: 0–2.5 min, 0% B; 2.5–7.5 min, linear ramp to 20% B min, 7.5–13 min, linear ramp to 45% B; 13–21 min linear ramp to 99% B and held at 99% B until 25 min. At 25 minutes, the 10-port valve was switched to reverse flow (back-flush) through the column, and the solvent composition changed to 95% C and held there for 3 min. From 28 to 28.5 min, the flow rate was ramped to 0.8 mL/min, held until 32.5 min, then reduced to 0.6mL/min. From 32.5 to 33.25 the solvent was ramped from 99–0% C while flow was simultaneously ramped down from 0.6–0.4mL/min and held until 39.9 min., at which point flow rate was returned to starting conditions at 0.25mL/min. The 10-port valve was returned to restore forward flow through the column at 40 min. An isocratic pump was used to introduce reference mass solution through the reference nebulizer for dynamic mass correction. Total run time was 30 min. The injection volume was 5μL. Data was analyzed using MassHunter ProFinder 8.0 software (Agilent Technologies).

### Tumor expression data

mRNA-seq Z-score expression and patient survival data from Mackay et al. (2017) was obtained using PedCBioPortal (Children’s Hospital of Philadelphia Research Initiative)(2, 30, 31). Data was filtered for patients who had confirmed WT Histone H3 or H3K27M mutant (*H3F3A* or *HIST1H3B*) and the Z-score for each gene of interest was extracted and sorted based on mutational status. Statistical analyses were performed using a two-tailed t-test in GraphPad Prism 9.0.

### Long-term neurosphere assay and live cell imaging

H3K27M cells (3×10^5–4×10^5) were cultured in 6-well dishes for 48hrs until they formed small spheres which were then treated with mycophenolic acid (MPA) for 6hrs prior to irradiation in the previously described apparatus. Approximately 18hrs later, spheres were dissociated and replated at low density (500 cells/well) in 96-well plates. Plates were incubated for 5–10 days in a BioSpa incubator (Agilent Technologies) and imaged every 24hrs in a Cytation 5 plate reader with the 4x objective. Gen5 software (Agilent Technologies) was used to analyze the data. A 2D area threshold of 2600um^2 was used to pick spheres, approximating the 50-colony threshold used in standard clonogenic survival assays. End-point sphere numbers were used to calculate the surviving fraction of cells. The Dmid of the 0μM MPA control was divided by the Dmid of the MPA-treated groups to calculate the Enhancement Ratio (ER) to display changes in RT efficacy.

### Stereotactic orthotopic implantation

Rag1-KO C57BL/6 mice were anesthetized and provided carprofen analgesic before removing the scalp fur and sterilizing the incision site. An incision was made along the midline of the scalp and a small hole in the skull was made using an electric burr hole drill or handheld drill bit at coordinates 2–3mm lateral, 0.5–1mm rostral or caudal (depending on the injection rig used) from the bregma. Approximately 1.5×10^5–2.0×10^5 cells (~ 5.0×10^4 cells/μL) in a volume of 3μL were implanted in the cortex at depth of approximately 2.5mm using 10μL Hamilton syringes. After injections, wounds were sutured and were given triple antibiotic ointment. Atipamezole was given reverse the anesthetic in heated cages. Mice were given diet gel supplement and monitored for 10 days, including a second dose of carprofen the day following the surgery. For some experiments, a PhD Ultra multi-syringe pump (Harvard Apparatus) was utilized to facilitate simultaneous implantation of up to 8 mice at once. A minimum of 6 mice were used per treatment group in subsequent *in vivo* experiments.

### Bioluminescent imaging (BLI) and mouse treatment

Mice were administered sterile-filtered 30mg/mL luciferin solution (Syd Laboratories) via IP injection. Ten minutes post-injection, BLI signal was measured using an IVIS Spectrum imaging station and Luminescent Flux Values were obtained for each tumor. BLI flux values were used to randomize mice into four treatment groups (10 mice/group) based on BLI flux average signal. BLI was repeated 1–2x weekly during efficacy studies. Treatment groups included: Vehicle Control (0.5% w/v methylcellulose, 0.1% Tween-80 in ddH2O, mycophenolate mofetil (MMF) alone, RT alone, and MMF + RT. MMF (150mg/kg) was administered by oral gavage for 11 consecutive days starting between 13–18 days post-implantation. Mice were sedated using 2.5% isoflurane and RT was administered in 2Gy fractions on days 2, 4–5, 8–9, and 11 of MMF treatment using an Orthovoltage irradiator. Mice were euthanized upon development of neurological symptoms and survival data was analyzed using the Kaplan-Meier method using GraphPad Prism 9.0.

## Results

### The H3K27M mutation confers global changes to cellular metabolism.

**Figure 1:**
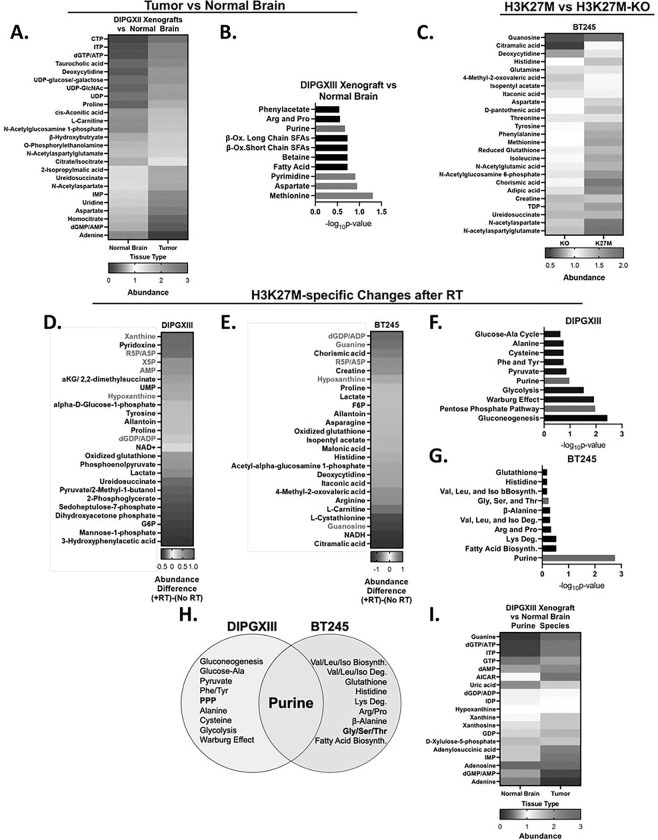
The H3K27M mutation influences metabolic phenotypes and the metabolic response to radiation. *A.)* Untreated intracranial DIPGXIII xenografts and contralateral normal brain issue (n=2 mice/group) were harvested, and their metabolites were collected by methanol extraction and measured using LC/MS. Data are presented as the top 25 significantly different metabolites between Tumor and NB and are ordered by difference in average median centered abundance between K27M and KO cells (K27M-KO). *B.)* Metabolite set enrichment analysis of all metabolites whose abundance were significantly different between Tumor and normal brain (83 total). Blue bars indicate pathways important for DMG-H3K27M biology as reported in the literature. Red bars indicate pathways important for purine metabolism. *C.)* Metabolites levels in untreated BT245 isogenic cell lines as measured using LC/MS. Data represent the top 25 significantly different metabolites between *H3F3A* KO and K27M cells. Data are ordered by difference in median centered abundance in descending order. Metabolite extractions were performed in triplicate. *D. and E.)* H3K27M isogenic cell lines DIPGXIII *(D.)* and BT245 *(E.)* were treated with or without 4Gy RT and harvested for metabolite quantification 2hrs later. Metabolites meeting the FC threshold were selected. Data represent the top 25 largest absolute post-RT abundance differences between H3K27M and *H3F3A* KO cells in descending order. Red indicates abundances over a post-RT FC value of 0.0. Blue indicates abundances under a post-RT FC value of 0.0. *F. and G.)* Metabolic set enrichment analysis was performed on the metabolite lists for DIPGXIII *(D.)* and BT245 *(E.)* and ordered based on −log_10_p-value. Red bars indicate pathways important for purine metabolism. *H.)* Venn Diagram depicting the metabolic pathways enriched in RT-treated DMG-H3K27M isogenic cell lines. Bold-faced font indicates metabolic pathways important for purine metabolism and synthesis. *I.)* Purine metabolites from DIPGXIII xenograft tumors and contralateral normal brain from *A.)* were compared and ordered by difference in average median centered abundance between tumor and normal brain.

Altered cellular metabolism is a hallmark of cancer(32). To begin to understand how the H3K27M mutation alters metabolism and facilitates treatment resistance, we quantified how metabolite levels differed between normal brain and DMG-H3K27M xenograft tumor tissue. Using fluorescence-guided microdissection, we separated GFP and luciferase-expressing DIPGXIII (DIPGXIII-GFP/LUC) orthotopic tumors from surrounding normal brain and quantified their metabolites. The brain-specific metabolite N-cetylaspartate was higher in cortex compared to DMG tumors, indicating that our fluorescent-based separation was successful (Fig S1A). Numerous other metabolites including dGTP/ATP, citrate/isocitrate, and UDPglcNAC were elevated in DMG while adenine, ureidosuccinate, and aspartate were lower indicating that DMG tumor tissue possesses a distinct metabolome compared to normal brain (Fig. 1A). To understand the biology of these dysregulated metabolites, we performed metabolic pathway enrichment analysis and found enrichment of pyrimidine and methionine metabolism in DMG-H3K27M tumors (blue bars), consistent with recent reports of the importance of these pathways in H3K27M tumors, as well as purine metabolism (Fig. 1B)(25, 26, 29).

Some of these metabolic alterations might be caused specifically by the H3K27M mutation(24) while others might be related to alternative mutations or oncogenic transformation in general. To understand how the H3K27M mutation specifically affected metabolism, we used isogenic patient-derived DMG-H3K27M cell line pairs (DIPGXIII and BT245) in which the H3K27M mutation has been removed from the parental cell line using CRISPR/Cas9 (DIPGXIII-KO and BT245-KO)(20). We confirmed the presence of the mutation in parental cells and the corresponding lack of H3K27me3 (Fig S1B). We quantified metabolites in these isogenic pairs using LC/MS and found that H3K27M-expressing cells have an altered metabolome compared to H3K27M-KO counterparts (Fig. 1B, S2, and Supplemental Table 1). These findings are consistent with prior studies and confirm that the oncohistone plays an active role in altering DMG metabolism(24).

### RT induces changes in purine metabolism in H3K27M cells.

We hypothesized that the H3K27M mutation influences the metabolic response to RT, thereby conferring RT resistance(33–35). Using the H3K27M-isogenic models, we performed steady-state metabolomics on untreated and RT-treated cells two hours after RT to capture early shifts in cellular metabolism that might mediate RT resistance (Fig. 1D–E). We identified numerous metabolites that changed following RT in either the H3K27M or H3K27M-KO cells (Fig. 1D–E and Supplemental Table 2). Metabolites like glutamine in the DIPGXIII cells and aspartate in the BT245 model had similar responses to RT in both H3K27M and H3K27M-KO cells (Fig S3A-B). Other metabolites like xanthine in the DIPGXIII model and dGDP/ADP in the BT245 model had responses to RT that varied depending on the presence of the H3K27M mutation (Fig S3C-D, Supplemental Table 2).

To better understand which metabolic changes were H3K27M-specific, we calculated the differences in fold-change (FC) following RT between H3K27M and H3K27M-KO cell lines for each metabolite (Fig. 1D–E and Supplemental Table 2). We noticed that numerous purine species including xanthine, hypoxanthine, guanine, AMP, and ADP/dGDP responded differently to RT in H3K27M mutant cells compared to KO controls (Fig. 1D–E, Supplemental Table 2). Metabolite set enrichment analysis of the top 25 metabolites highlighted purine metabolism in both H3K27M-isogenic cell line pairs (Fig. 1F–G, H). Other purine metabolism-related pathways found included the pentose phosphate pathway (DIPGXIII) (Fig. 1F) that creates the ribose-5-phosphate sugars needed for nucleotides, and glycine/serine/threonine metabolism (BT245) that generates metabolites needed for purine ring construction (Fig. 1G). We reanalyzed DIPGXIII-GFP/LUC tumor data to focus on purine species and found that tumor tissue has an inverse purine metabolic phenotype to that of the normal tissue (Fig. 1I). Interestingly, our group has shown that purine metabolism mediates treatment resistance in adult glioblastoma (GBM), another form of astrocytoma(16). Together, these observations suggest that purine metabolism changes following RT in an H3K27M-specific fashion.

### Purine metabolic flux and enzyme expression are influenced by the H3K27M mutation.

**Figure 2:**
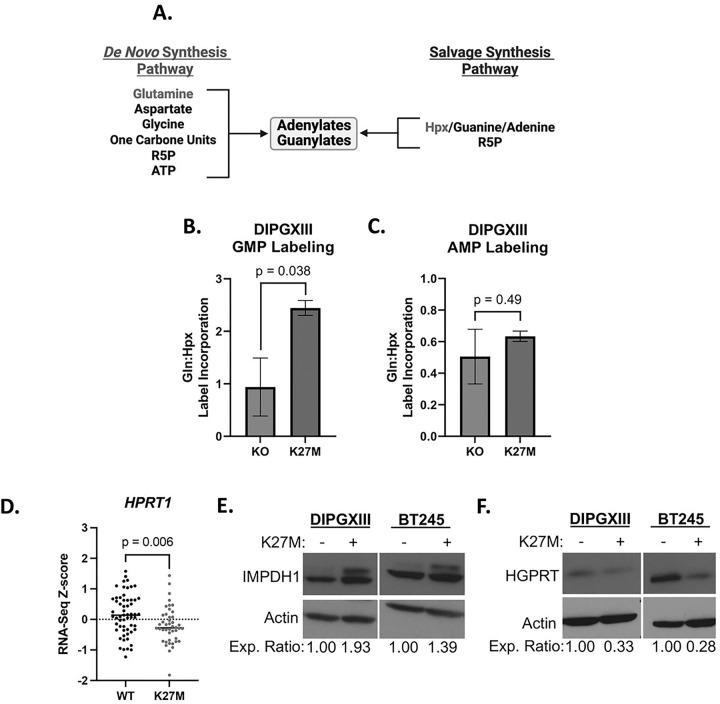
Increased reliance on *de novo* purine synthesis in H3K27M cells. *A.)* Schematic of purine synthetic metabolic pathways showing where the purine *de novo* and salvage pathways converge to form guanylate purines from IMP and free purine bases, respectively. *B*. and *C.)* Ratio of ^15^N-glutamine:2D-Hpx stable isotope tracer label incorporation into GMP *(B.)* and AMP *(C.)* in untreated DIPGXIII H3K27M-isogenic cell lines. *D.)* Publicly available RNAseq Z-score data for *HPRT1* transcript expression from pediatric high-grade gliomas (pHGG) was obtained through PedCBioPortal and filtered to include only samples with known H3 mutational status (for both *H3F3A* and *HIST1H3B*) to include all known H3WT (n=59)and combined H3K27M (H3F3A-mut+*HIST1H3B*-mut) samples (n=44) *E.) and F.)* Immunoblots of HGPRT and IMPDH1 *(E.)* and HGPRT *(F.)* expression in DMG-H3K27M isogenic paired cell lines. Densitometric values were calculated using ImageJ software and expression values were normalized to H3K27M-KO cells. Expression ratios are listed below the blot images. Statistical analyses were performed using two-tailed t-tests.

We and others have found that purine species, especially guanylates, contribute to treatment resistance in brain tumors(16, 36). We reasoned that targeting purine synthesis could increase RT efficacy in DMG-H3K27M. Purine nucleotides are produced through either the *de novo* or the salvage synthesis pathways (Fig. 2A). To determine which of these pathways DMG-H3K27M tumors may rely on, we performed stable isotope tracing to measure their baseline flux. This technique utilizes nutrients carrying one or more nonradioactive atoms that are heavier than the naturally occurring form, allowing for their detection by mass spectrometry. Heavy isotope labeling patterns in metabolites formed from the tracer molecule then allows us to track how cells change their metabolic fluxes in response to RT. We tracked *de novo* synthesis (DNS) using ^15^N-glutamine as it is critical for the formation of newly synthesized purine rings (Fig S4B). Purine salvage was measured using 2D-Hpx that is converted into the common purine precursor, IMP, which can then be converted into GMP or AMP (Fig S4A). Here, we observed that DIPGXIII H3K27M-expressing cells have a significantly higher ratio of ^15^N-glutamine:2D-Hpx labeling of GMP than do H3K27M-KO cells, indicating that H3K27M cells prefer DNS to create GMP (Fig. 2B). This is further evidenced by higher ^15^N-glutamine labeling (Fig S4C) and lower 2D-Hpx labeling (Fig S4E) and of GMP in H3K27M cells than in H3K27M-KO cells. There was no H3K27M-specific preference for DNS-derived AMP (Fig. 2C, S4D and F).

Given the H3K27M-specific differences in GMP synthesis, we assessed the expression of the rate limiting enzymes in both *de novo* guanylate synthesis (IMPDH1 and IMPDH2) and guanylate salvage (HGPRT). Using publicly available RNAseq data from patients bearing pediatric high-grade gliomas (pHGG)(2), we found that H3K27M pHGG tumors expressed less *HPRT1*, which encodes the rate-limiting guanylate salvage enzyme HGPRT (Fig. 1D). At the protein level, we observed increased total expression of IMPDH1 protein (Fig. 2E) and decreased total expression of HGPRT (Fig. 2F) in H3K27M cells compared to H3K27M-KO counterparts. We found no difference in the expression of *IMPDH1* or *IMPDH2* between H3WT or H3K27M pHGG tumors (Fig S5A-B). We also did not observe a difference in the expression of *GMPS*, the gene-encoding the enzyme downstream of *IMPDH1/2* (Fig S5C).

**Figure 3:**
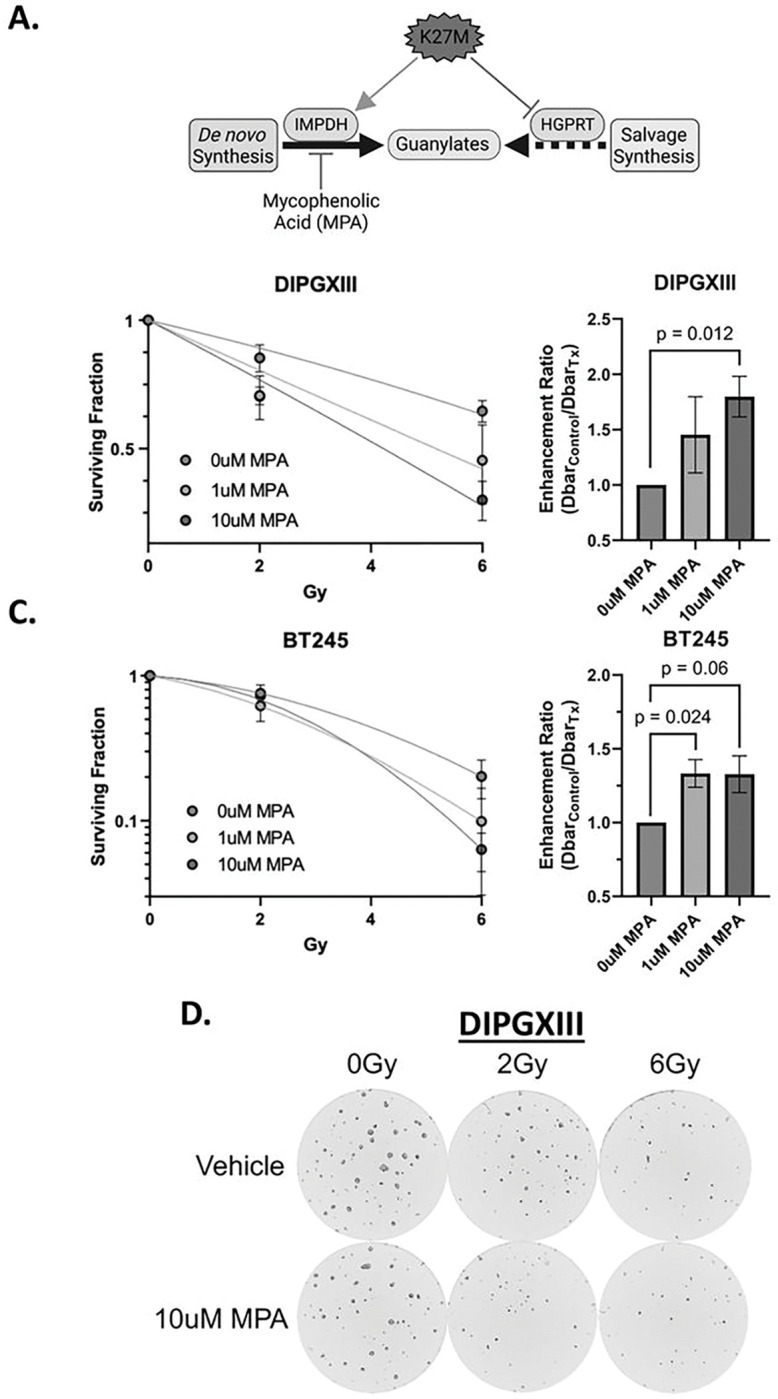
*De novo* purine synthesis inhibition radiosensitizes K27M cells. *A.)* Schematic depicting the hypothesis that the K27M mutation induces defective guanylate purine salvage through HGPRT suppression, leaving K27M cells vulnerable to *de novo* guanylate synthesis using IMPDH inhibition using MPA. *B. and C.)* Long-term neurosphere growth assays for DIPGXIII *(B.)* and BT245 *(C.)* H3K27M-expressing cell lines treated with increasing doses of RT (0, 2, 6Gy) with or without 1–10mM MPA (*left*) and corresponding enhancement ratio (Dbar_control_/Dbar_Tx_) for each concentration of MPA (*right*) administered with RT. Each long-term neurosphere assay was performed 3x per cell line. Statistical analyses were performed using two-tailed t-tests. *D.)* Live cell imaging analysis of DIPGXIII neurospheres treated with 0–6Gy +/− 10mM MPA. Images taken 12 days after replating in a 96-well plate. Images acquired using a Cytation 5 plate reader and attached BioSpa incubator (Agilent Technologies).

Together, these results suggest that H3K27M cells inactivate guanylate salvage due to decreased expression of HGPRT and have increased reliance on *de novo* guanylate synthesis that is facilitated by increased IMPDH protein expression (Fig. 3A). Consistent with our tracing results, we did not observe similar patterns in the enzymes used to synthesize adenylates or in those used in the upstream steps of DNS (Fig S5D-F).

### De novo guanylate synthesis inhibition increases RT efficacy in H3K27M models.

Increased reliance on *de novo* guanylate synthesis in H3K27M expressing cells suggests that inhibition of this pathway may have utility as monotherapy(37) or in combination with RT (Fig. 3A). Inhibition of *de novo* guanylate synthesis using the IMPDH inhibitor mycophenolic acid (MPA) radiosensitizes adult GBM brain tumors and is being evaluated in a Phase 0/1 clinical trial (NCT04477200) in human GBM patients(16, 38). To explore a similar combination strategy in H3K27M cells, we treated DIPGXIII and BT245 patient-derived H3K27M-expressing cells with 0–10uM MPA in combination with increasing doses of RT (0–6Gy) to determine its effect on radiosensitivity. By measuring neurosphere formation over time, we observed dose-dependent decreases in the surviving fraction of DIPGXIII and BT245 neurospheres following RT which was augmented upon addition of MPA at either concentration (1μM and 10μM), leading to increased RT enhancement ratios (DIPGXIII: 1.45 and 1.78, BT245: 1.33 and 1.33) (Fig. 3B–C). This can be observed visually where we see that combined RT and MPA reduces neurosphere size and number in both of our H3K27M-expressing models, even at a low dosage of radiation (Fig. 3D, S6). Interestingly, we and others have observed single agent efficacy of MPA in DMG-H3K27M cells where we see reduced sphere size and number with MPA alone (Fig. 3D, S6). This suggests that DMG-H3K27M cells were more dependent on DNS at baseline, consistent with our isotope tracing studies and with previous reports(39). Taken together, these results show that IMPDH inhibition might help overcome RT resistance in H3K27M-expressing cells.

**Figure 4:**
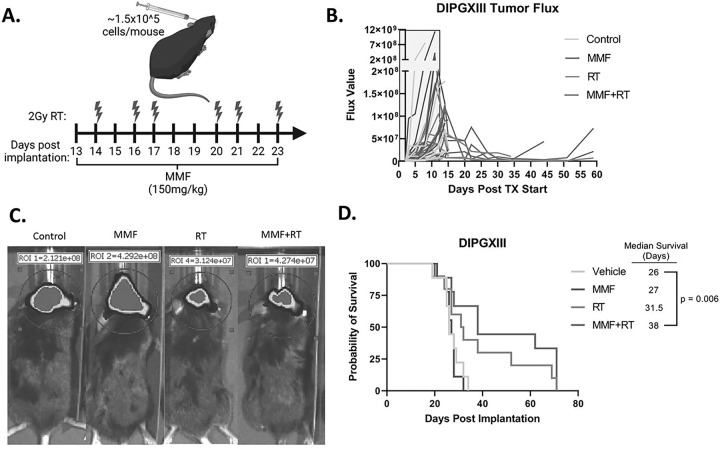
Combination RT and *de novo* purine synthesis inhibition reduces DMG-H3K27M tumor size and extends mouse survival in vivo. *A.)* Schematic of treatment schedule administered to DIPGXIII-bearing Rag1-KO mice. DIPGXIII cells expressing GFP and Luciferase (LUC) (DIPGXIII-GFP/LUC) were orthotopically implanted into the cortex and mice were administered 150mg/kg MMF for 11 days, with 6 intermittent doses of 2Gy RT (red bolts). *B.)* Spider plot of bioluminescent signal flux values for individual mouse tumors. Blue box indicates the treatment period. *C.)* Representative bioluminescent signal images for one mouse per treatment group 24 days after implantation. *D.)* Kaplan-Meier survival analysis of Rag1-KO mice bearing treated and untreated DIPGXIII-GFP/LUC orthotopic xenograft tumors (n=10 mice/group). Statistical analysis was performed by comparing individual survival curves of individual treatment groups in GraphPad Prism 9.0.

With these promising *in vitro* results, we next wanted to test the *in vivo* efficacy of RT in combination with IMPDH inhibition in H3K27M expressing tumors. Previous work from our group and others has shown that mycophenolate mofetil (MMF), the pro-drug of MPA, has efficacy in intracranial adult GBM models(16) and we sought to employ a similar strategy here. DIPGXIII-GFP/LUC cells were orthotopically implanted into the cortex and monitored by bioluminescent imaging (BLI). Once tumors were detectable, mice were randomized and treated with RT alone, MMF alone, combined RT and MMF, or vehicle control (Fig. 4A). Mouse weight was largely unaffected by the treatment course (Fig S7). Unlike our *in vitro* experiments, MMF alone had little effect on tumor size measured by BLI (Fig. 4B). Both RT and MMF + RT decreased tumor bioluminescence, but in both groups, tumors eventually regrew, and we did not observe marked difference between the two groups (Fig. 4B–C). MMF alone had no effect on median survival versus vehicle controls (27d vs 26d, respectively, *p* = 0.6). RT alone increased mouse survival over vehicle control (31.5d), but not in a statistically significant manner. Combination MMF + RT significantly extended survival over vehicle controls (38d vs 26d, *p* = 0.006), but did not cure tumors (Fig. 4D). These findings suggest that while combination MMF + RT treatment extends survival, there may be resistance mechanisms employed by DMG-H3K27M tumors to evade MMF + RT treatment.

### H3K27M cells upregulate purine salvage in response to RT.

**Figure 5:**
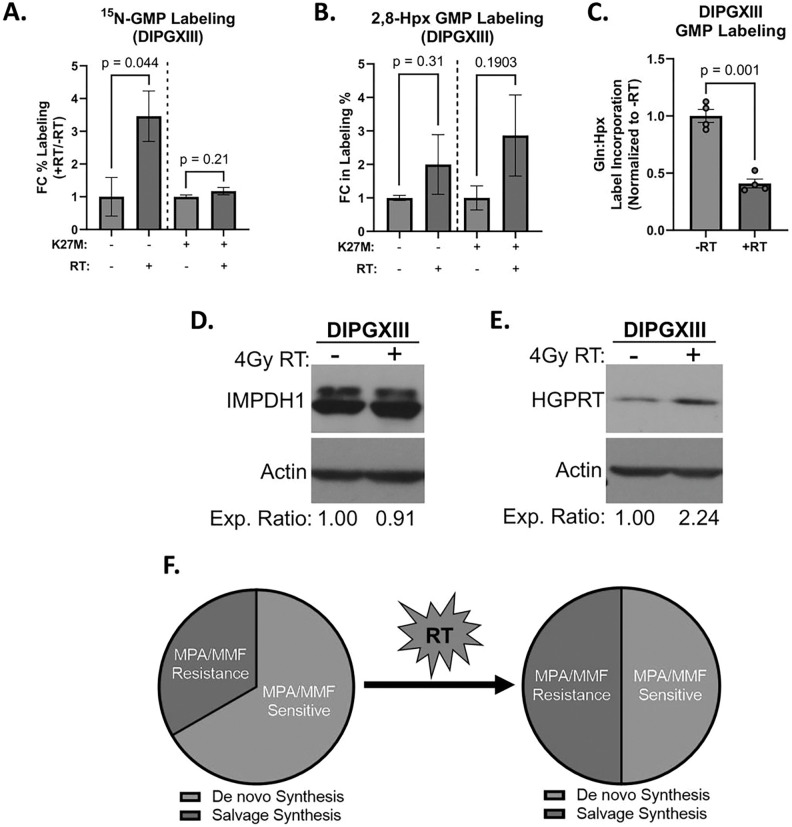
H3K27M-expressing cells increase purine salvage following RT. *A.)* Fold change of the percentage of total GMP in DIPGXIII H3K27M-isogenic cell lines labeled by ^15^N-glutamine tracer before (gray bars) and 3hrs after 4Gy RT (red bars). Data are normalized to untreated control samples. *B.)* Percent of total GMP in DIPGXIII H3K27M-isogenic cell lines labeled by 2D-Hpx tracer before (gray bars) and 3hrs after 4Gy RT (red bars). Data are normalized to untreated control samples. *C.)* Ratio of Gln:Hpx label incorporation into GMP after 3 hours in DIPGXIII cells that were either untreated or treated with 4Gy RT. Data is normalized to untreated control. *D.)* Immunoblot of IMPDH expression before and after 4Gy RT in DIPGXIII cells. Densitometric analysis was performed using ImageJ software and expression values were normalized to No-RT controls. Values are listed below the blot images. *E.)* Immunoblot of HGPRT expression before and after 4Gy RT in DIPGXIII cells. Densitometric analysis was performed using ImageJ software and expression values were normalized to No-RT controls. Values are listed below the blot images. *F.)* Schematic depicting the RT-induced shift in DNS and salvage flux that leads to a loss of MPA sensitivity. Statistical analyses were performed using two-tailed t-tests in GraphPad Prism 9.0.

We reasoned that DMG-H3K27M tumors might upregulate purine metabolism in response to RT and limit the efficacy of MMF *in vivo*. We employed stable isotope tracing to measure the activity of both *de novo* and salvage purine synthesis following RT (Fig. 5, S8). We showed previously that without RT, H3K27M cells converted more glutamine-derived ^15^N into GMP but not AMP, than did H3K27M-KO cells (Fig. 2B, S4E-F). RT increased ^15^N incorporation into GMP in H3K27M-KO cells, but H3K27M cells showed no change (Fig. 5A). We also observe decreased ^15^N-labeled AMP in both H3K27M and H3K27M-KO cells, though this effect is greater in H3K27M-KO cells (Fig S8A). These findings suggest that H3K27M cells do not increase DNS following RT.

Previously we found that H3K27M cells converted less hypoxanthine into GMP than did H3K27M-KO cells at baseline, which suggested that the H3K27M mutation slows guanylate salvage (Figs. 2B, 3A). Unexpectedly, both H3K27M cells and H3K27M-KO cells *increased* hypoxanthine salvage into GMP post-RT (Fig. 5B). This leads to a roughly sixty percent reduction in ratio of Gln:Hpx incorporation into GMP, highlighting increased reliance on salvage synthesis after RT in H3K27M cells (Fig. 5C). There was no change in hypoxanthine-derived AMP in either H3K27M or H3K27M-KO cells (Fig S8B). The ability for H3K27M cells to increase guanylate salvage following RT could account for their resistance to MMF + RT treatment.

To understand how H3K27M cells can upregulate purine salvage but not DNS following RT, we examined the expression of the rate limiting enzymes in these pathways. Consistent with an inability to upregulate DNS, H3K27M cells did not show increased IMPDH1 protein expression following RT (Fig. 5D). However, RT increased HGPRT expression in H3K27M-expressing DIPGXIII cells (Fig. 5E). Thus, while H3K27M mutant cells appear to rely on MPA-sensitive DNS in the unperturbed state, they upregulate MPA-resistant purine salvage synthesis following RT, which may mediate resistance to DNS inhibitors like MMF *in vivo* (Fig. 5F).

### HPRT1 loss leads to extended survival in mice bearing DMG-H3K27M xenografts.

**Figure 6:**
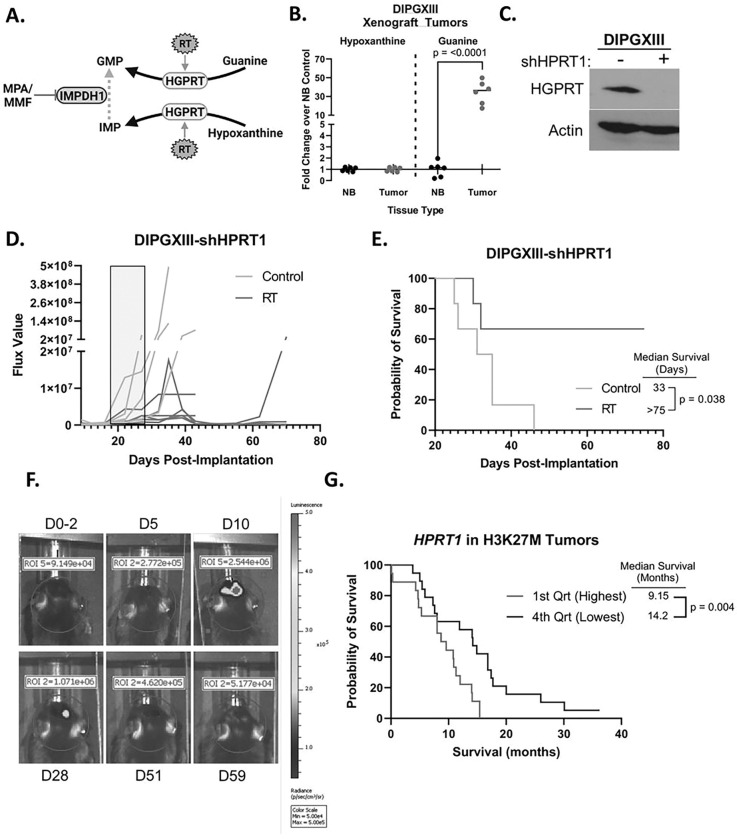
Genetic inhibition of purine salvage in H3K27M-expressing tumors potentiates the effects of RT. *A.)* Schematic showing mechanism of *in vivo* resistance to MMF via increased intratumoral guanine abundance. *B.)* Hypoxanthine and guanine abundances in orthotopic DIPGXIII-GFP/LUC xenograft tumors and contralateral normal brain tissue. Tissues were harvested by GFP fluorescence-guided mechanical resection, and their metabolites collected via methanol extraction before LC/MS. Data is normalized to normal brain (NB). *C.)* Immunoblot of HGPRT in DIPGXIII-GFP/LUC-shHPRT1 cells transfected with pooled shHPRT1 lentivirus and selected with puromycin. *D.)* Spider plot of bioluminescent signal flux values for individual mouse tumors. Blue box indicates the treatment course. *E.)* Kaplan-Meier survival analysis of Rag1-KO mice bearing DIPGXIII-GFP/LUC-shHPRT1 orthotopic xenograft tumors given vehicle control or RT (n=7 mice/group). Statistical analysis was performed by comparing individual survival curves using GraphPad Prism 9.0 software. *F.)* Bioluminescent imaging of an RT-treated mouse bearing an orthotopic DIPGXIII-GFP/LUC-shHPRT1 xenograft tumor that experienced a complete response following treatment. D# indicates the day at which the image was taken with respect to the end of the treatment regimen. The first image was taken two days prior to the completion of the treatment regimen (D0–2). *G.)* Kaplan-Meier survival analysis of patient H3K27M-expressing tumors based on HPRT1 expression where the 1^st^ quartile represents the highest *HPRT1* expression, and the 4^th^ quartile represents the lowest *HPRT1* expression.

HGPRT can salvage both hypoxanthine and guanine (Fig. 3A). Salvaged hypoxanthine is converted to IMP whose conversion into GMP is blocked by MPA/MMF (Fig. 6A). Salvaged guanine, by contrast, directly forms GMP and bypasses IMPDH1 inhibition. Thus, upregulation of guanine salvage would cause MMF resistance while upregulation of hypoxanthine salvage would not, though both could be mediated by increased HGPRT expression. Our initial neurosphere assays (Fig. 3) were performed in TSM media where hypoxanthine is the dominant available purine base(40). However, the mouse tumor microenvironment is vastly different than the cell culture dish regarding metabolite availability. To determine which bases were available for guanylate salvage in DMG-H3K27M tumors, we analyzed both guanine and hypoxanthine levels in orthotopic DIPGXIII-GFP/LUC tumors and contralateral cortex. While DIPGXIII-GFP/LUC tumors and normal brain contained roughly the same abundance of hypoxanthine, tumor tissue possessed a 34-fold higher abundance of guanine (Fig. 6B). High intratumoral guanine coupled with increased salvage activity following RT (Figs. 5B) and RT-induced increases in HGPRT expression (Fig. 5D) suggest that HGPRT-mediated salvage of guanine can bypass IMPDH1 inhibition in H3K27M cells, leading to continued RT resistance (Fig. 6A) despite MMF treatment.

We then wanted to determine if HGPRT inhibition could increase RT efficacy in DMG-H3K27M xenograft tumors. No blood-brain barrier (BBB)-penetrant HGPRT inhibitors currently exist, so we utilized a pooled shRNA to knockdown *HPRT1* expression in DIPGXIII-GFP/LUC cells (DIPGXIII-GFP/LUC-shHPRT1) (Fig. 6C). These cells were then implanted into the cortices of Rag1-KO mice. Tumor-bearing mice were RT-treated as previously described (Fig. 4A). Mouse weight was largely unaffected by the treatment regimen (Fig. S8). RT alone greatly reduced BLI signal in mice bearing DIPGXIII-GFP/LUC-shHPRT1 tumors (Fig. 6D). Median survival in DIPGXIII-GFP/LUC-shHPRT1 tumors was similar to vehicle control mice in our first experiment (33d vs 26d), suggesting that purine salvage is dispensable for initial tumor growth (Fig. 4D and 6E). However, irradiation of HGPRT-deficient tumors significantly extended survival (> 75d) and led to multiple complete responses (Fig. 6E–F). Lastly, using publicly available patient tumor data, patients bearing H3K27M tumors had an inverse correlation between median survival and *HPRT1* expression (Fig. 6G)(2). Together, these findings indicate that purine salvage through HGPRT may mediate RT resistance in DMG-H3K27M.

## Discussion

In this study, we have defined new metabolic strategies to overcome treatment resistance in devastating DMG-H3K27M tumors. Using steady-state and stable isotope tracing metabolomics in patient-derived H3K27M-isogenic cell lines, we found that H3K27M cells preferentially rely on the DNS of guanylates. This reliance is likely due to low HGPRT and high IMPDH1 expression in H3K27M cells. Inhibiting DNS of guanylates potentiated the effects of RT on H3K27M models *in vitro* and *in vivo* but did not cure tumors. Intriguingly, H3K27M cells upregulated guanylate salvage in response to RT, and DMG-H3K27M xenografts had abundant intratumoral guanine. This suggested that RT-induced guanylate salvage might be an adaptive mechanism of RT resistance. Consistent with this model, inhibition of guanylate salvage in DMG-H3K27M tumors overcame RT resistance and led to complete responses in several mice. Together, our findings suggest that DMG-H3K27M tumors rely on both *de novo* and salvage guanylate synthesis, with salvage preferentially contributing to radiation resistance.

Our findings add to the growing body of work showing that altered metabolism is a targetable vulnerability in DMG. These tumors cells preferentially rely on *de novo* pyrimidine synthesis and methionine consumption(25, 26). Like others(39), we have found that DMG-H3K27M tumors also rely on *de novo* guanylate synthesis for survival. However, none of these seminal findings have considered how H3K27M-mediated metabolic changes are related to RT resistance. Here, we unexpectedly discovered that activation of guanylate salvage may be an adaptive mechanism to resist RT. Thus, our work suggests that while strategies targeting methionine, pyrimidine, or *de novo* guanylate synthetic metabolism may be effective as monotherapies, targeting radiation-induced guanylate salvage in combination with RT may be an effective combination strategy to combat DMG-H3K27M tumors.

Our discoveries have important implications for the treatment of DMG-H3K27M patients. Nearly every patient with DMG-H3K27M receives RT, so a drug that selectively overcomes DMG-H3K27M RT resistance by inhibiting guanylate salvage could immediately be tested in combination with RT in patients. In this study, we utilized a genetic approach to inhibit guanylate salvage, which cannot readily be translated to patients. 6-mercaptopurine (6-MP) is an HGPRT inhibitor that is effectively used in acute lymphoblastic leukemia (ALL) patients to maintain remission and has been shown to induce apoptosis in renal cancer cells(41, 42). However, it was shown to have poor BBB penetrance in preclinical studies(41). Acyclic nucleoside phosphonates (ANPs) are being investigated as antimalarial compounds that target *Plasmodium spp*. HGPRT/HG(X)PRT(43). These ANPs were shown to complex with human HGPRT, however it is unknown if they can cross the blood-brain barrier. Preclinical studies to test the pharmacokinetics of molecules like ANPs and drug discovery directed at improving the BBB penetrance of existing inhibitors like 6-MP or development of new molecules could yield new strategies to target RT-induced guanylate salvage in DMG-H3K27M tumors.

These studies present lingering questions that still need to be answered. While we know that RT induces HGPRT expression, we do not know the molecular mechanism behind this upregulation. The rapid change in HGPRT expression (< 3hrs) suggests an epigenetic mechanism that could be related to the underlying H3K27M mutation. Additionally, we do not know if the ratio of DNS to salvage changes further as time progresses. We also do not know the source of the abundant guanine in our xenograft model, which could be due to high turn-over from malignant cells or secretion from non-cancerous cells such as atrocytes(39, 44). Lastly, we do not know if the therapeutic window for inhibiting guanylate salvage will be large enough between normal brain and DMG-H3K27M tissue to improve treatment efficacy. The normal brain salvages purines and HGPRT loss underlies Lesch-Nyan disease, though it is possible that temporary inhibition of HGPRT in normal tissues may be tolerated(45).

## Conclusions

Together, our studies suggest that multiple routes of purine synthesis are important for DMG growth. Combining *de novo* purine synthesis inhibition with RT, an approach that we are utilizing clinically for adult patients with GBM(38), may therefore be less promising in DMG. Further preclinical study is needed to determine if combining small molecule inhibition of purine salvage with radiation can improve RT efficacy and be translated to clinical trials to improve DMG-H3K27M patient outcomes.

## Data Availability

Human tumor RNAseq Z score data and patient survival data were obtained from the Institute of Cancer Research (London, UK) using PedcBioPortal (Children’s Hospital of Philadelphia Research Initiative, https://pedcbioportal.kidsfirstdrc.org/) where it is currently available(2, 30, 31). All other data supporting the findings of this study can be made available upon request to the authors.

## Abbreviations

DMG: Diffuse midline glioma
DIPG: Diffuse intrinsic pontine glioma
H3K27M: Histone H3, K27M mutant
RT: Radiation therapy
HGPRT: Hypoxanthine guanine phosphoribosyl transferase
TSM: Tumor stem media
GFP/LUC: Green fluorescent protein/Luciferase
IMPDH: Inosine monophosphate dehydrogenase
LC/MS: Liquid chromatography/Mass spectrometry
2D-Hpx: 2,8-deuterium hypoxanthine
MPA: Mycophenolic acid
ER: Enhancement ratio
BLI: Bioluminescent imaging
MMF: Mycophenolate mofetil
GBM: Glioblastoma
DNS: *De novo* synthesis
BBB: Blood-brain barrier
NIH: National Institute of Health
NCI: National Cancer Institute
NINDS: National Institute of Neurological Disease and Stroke
